# Exploring the use of NIR and Raman spectroscopy for the prediction of quality traits in PDO cheeses

**DOI:** 10.3389/fnut.2024.1327301

**Published:** 2024-02-06

**Authors:** Giorgia Stocco, Laura G. Gómez-Mascaraque, Gaurav Kr Deshwal, Jordi Cruz Sanchez, Arnaud Molle, Valentina Pizzamiglio, Paolo Berzaghi, Georgi Gergov, Claudio Cipolat-Gotet

**Affiliations:** ^1^Department of Veterinary Science, University of Parma, Parma, Italy; ^2^Department of Food Chemistry and Technology, Teagasc Food Research Centre Moorepark, Fermoy, Ireland; ^3^Escola Universitària Salesiana de Sarrià, Barcelona, Spain; ^4^Consorzio del Formaggio Parmigiano Reggiano, Reggio Emilia, Italy; ^5^Department of Animal Medicine, Production and Health, University of Padova, Padova, Italy; ^6^Institute of Chemical Engineering, Bulgarian Academy of Sciences, Sofia, Bulgaria

**Keywords:** Parmigiano Reggiano, Grana Padano, NIR, Raman, Bayesian methods, Partial Least Squares, data fusion

## Abstract

The aims of this proof of principle study were to compare two different chemometric approaches using a Bayesian method, Partial Least Square (PLS) and PLS-discriminant analysis (DA), for the prediction of the chemical composition and texture properties of the Grana Padano (GP) and Parmigiano Reggiano (PR) PDO cheeses by using NIR and Raman spectra and quantify their ability to distinguish between the two PDO and among their ripening periods. For each dairy chain consortium, 9 cheese samples from 3 dairy industries were collected for a total of 18 cheese samples. Three seasoning times were chosen for each dairy industry: 12, 20, and 36 months for GP and 12, 24, and 36 months for PR. A portable NIR instrument (spectral range: 950–1,650 nm) was used on 3 selected spots on the paste of each cheese sample, for a total of 54 spectra collected. An Alpha300 R confocal Raman microscope was used to collect 10 individual spectra for each cheese sample in each spot for a total of 540 Raman spectra collected. After the detection of eventual outliers, the spectra were also concatenated together (NIR + Raman). All the cheese samples were assessed in terms of chemical composition and texture properties following the official reference methods. A Bayesian approach and PLS-DA were applied to the NIR, Raman, and fused spectra to predict the PDO type and seasoning time. The PLS-DA reached the best performances, with 100% correctly identified PDO type using Raman only. The fusion of the data improved the results in 60% of the cases with the Bayesian and of 40% with the PLS-DA approach. A Bayesian approach and a PLS procedure were applied to the NIR, Raman, and fused spectra to predict the chemical composition of the cheese samples and their texture properties. In this case, the best performance in validation was reached with the Bayesian method on Raman spectra for fat (R2VAL = 0.74). The fusion of the data was not always helpful in improving the prediction accuracy. Given the limitations associated with our sample set, future studies will expand the sample size and incorporate diverse PDO cheeses.

## 1 Introduction

European PDO (Protected Designation of Origin) cheeses are outstanding examples of historic inheritance, which is characterized by diversity and tradition in the world of dairy production. These cheeses, which include renowned types like Parmigiano Reggiano (PR), Grana Padano (GP), Roquefort, and Manchego, are praised for their flavors that are closely connected to their geographic origins. Behind the artisanal craftsmanship and centuries-old traditions of PDO cheese production lies a complex interplay of chemical and physical processes. Spectroscopic techniques have emerged as indispensable tools in the scientific field, providing invaluable insights into the composition, structure, and quality of these dairy products (1). Among these techniques, Near Infrared (NIR) and Raman spectroscopy are the most used in the food industry for the quantification of crucial cheese components such as moisture, fat, and protein content (2–4), facilitating precise quality control, and for the identification and quantification of specific compounds responsible for flavor and aroma (5). The top selling PDO cheeses in the world are the Italian PR and GP, with total export value exceeding $1.2 billion and $650 million USD, respectively (6). Despite their reputation in the global market, monitoring their quality is a complex task involving several challenges. Spectroscopic techniques, while valuable, face specific issues when applied to these cheeses, in part because obtaining representative samples from the cheese wheels is not always easy and because developing accurate calibration models for spectroscopic analysis requires extensive data collection and validation (7). Moreover, as both cheeses are vulnerable to counterfeiting and fraudulent replication, spectroscopic techniques used for authentication must be robust to detect subtle differences between authentic and imitation products.

When spectroscopy is used, the instrument is also an important factor, and its choice depends, apart from available resources, on the specific goals of the analysis, the cheese type, and the desired level of detail in terms of composition prediction. For example, NIR is well-suited for macronutrients, whereas Raman is more suitable for lipids, such as ester linkages in triglycerides and phosphodiester bonds in phospholipids. The presence of these functional groups results in unique Raman peaks, enabling the differentiation of the type of lipid (8–10). For these reasons, a combination of spectroscopic techniques has been proposed to improve the accuracy of composition trait predictions from cheese spectra (11–13). The performance of the models can be evaluated in different ways: use of latent variables, cumulative variance, standard error of calibration, standard error of cross validation, coefficient of determination, similarity map and salience dimension of common space, limit of detection, limit of quantification, linearity, model fit and uncertainties. The spectroscopic instrument and the chemometric approach used (and related setup of parameters and features) for predicting cheese composition or other traits of interest can impact the prediction accuracy. For example, while chemometric methods like Partial Least Squares Regression (PLSR), Principal Component Analysis (PCA), or Linear Regression models are more commonly used for the prediction of food (including cheese) composition and other traits (14), Bayesian approaches are less common but have gained attention in recent years due to their ability to handle complex data structures and to provide deeper insights into uncertainty and variable selection (15). However, the choice of chemometric method often depends on several factors, including also the expertise of the researchers.

Therefore, the aims of this study were to compare two different chemometric approaches using a Bayesian method and PLSR and PLS- PLS-Discriminant Analysis (PLS-DA) (i) for the prediction of the chemical composition and texture properties of the GP and PR PDO cheeses by using NIR and Raman spectra, (ii) and for their ability to distinguish between the two PDO and among their ripening periods.

## 2 Materials and methods

### 2.1 Experimental design

A total of 18 cheese samples were collected from 6 dairy plants comprised within the consortia of GP and PR dairy chain. Three dairy plants belonged to GP and three to PR PDO chains. For each dairy, three seasoning times were selected, and were 12, 20, and 36 months for GP and 12, 24, and 36 months for PR.

### 2.2 Collection of the spectra

**Figure 1:**
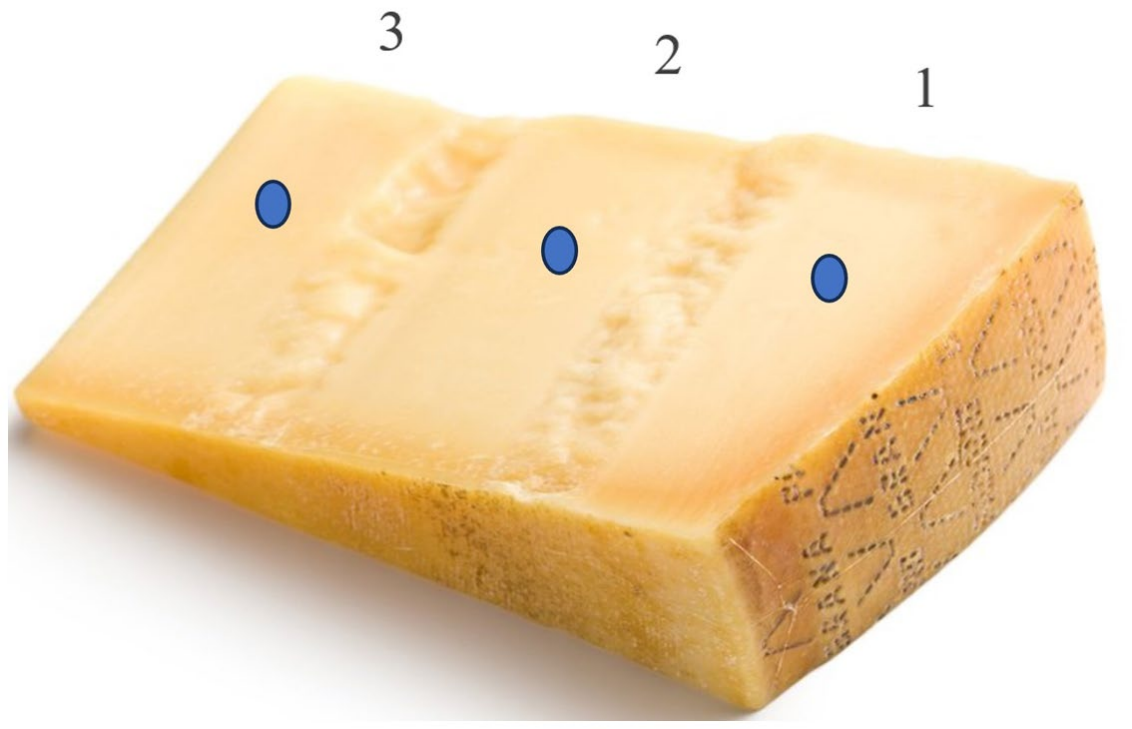
Example of a cheese sample analyzed and related spots (1: paste near the crust; 2: middle sample paste; 3: core of the entirecheese wheel).

**Figure 2:**
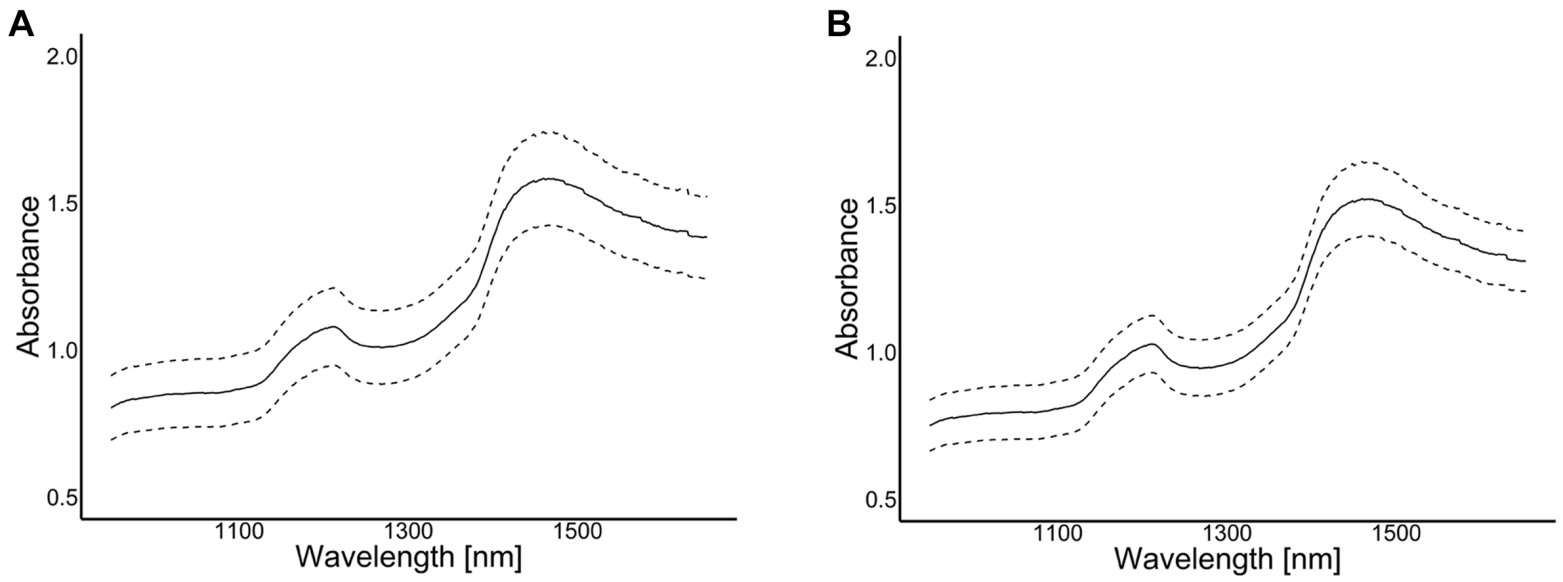
Absorbance spectra (the solid lines represent the average absorbance and the broken lines the mean ± 1 SD) of NIR portable instrument for Grana Padano (GP) **(A)**; Parmigiano Reggiano (PR) **(B)**.

A portable NIR instrument (Alba GraiNit, Padova, Italy), working within the spectral range 950–1,650 nm, was used on 3 selected spots of each cheese samplealong the radius of the cheese wheel (as indicated in Figure 1), for a total of 54 spectra collected. The spectra (average of 27 spectra within PDO type) are plotted in Figure 2A for Grana Padano and (Figure 2B) for Parmigiano Reggiano.

**Figure 3:**
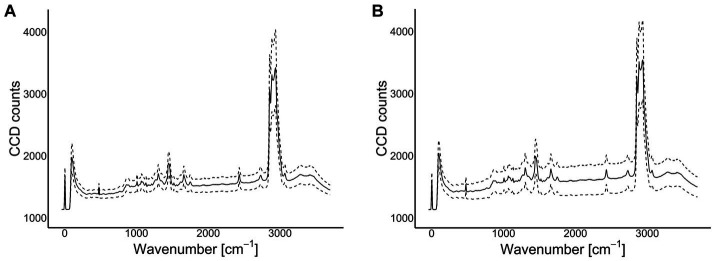
Absorbance spectra (the solid lines represent the average absorbance and the broken lines the mean ± 1 SD) of the Raman instrument for Grana Padano (GP) **(A)**; Parmigiano Reggiano (PR) **(B)**.

An Alpha300 R confocal Raman microscope (WITec, Ulm, Germany) was used to acquire the Raman spectra using a 532 nm laser at 40 mW and a 10x/0.25 objective. The integration time was 1 s and the number of accumulations 20. In this case, 10 individual spectra were collected for each cheese sample in each location (3 spots) for a total of 540 Raman spectra collected. The spectra (average of 270 spectra within PDO type) are plotted in Figure 3A for Grana Padano and (Figure 3B) for Parmigiano Reggiano.

### 2.3 Composition analyses

The chemical composition (moisture, protein, lipids) was analyzed on all the cheese samples. Briefly, the cheese samples were grinded (Thermomix TM6, Vorwerk), and approximately 2 g of grinded cheese sample was weighed and used for moisture and fat analysis using the microwave moisture analyzer (Smart 5 Turbo) and Nuclear Magnetic Resonance (NMR) fat analyzer (CEM Corporation). The microwave moisture analyzer applies microwave radiation to the sample, causing the water molecules to heat up and evaporate. The moisture content is then determined by measuring the weight loss of the sample before and after the microwave treatment. For the determination of fat, the NMR can distinguish the signal produced by hydrogen protons present in fats from that produced by all other sources of protons in food matrices, such as carbohydrates and proteins. The instrument is validated according to the AOAC regulations [Peer-Verified Method 1:2004 according to (16)]. The protein content of cheese samples was determined by Kjeldahl method (17). The steps typically involve digestion of the sample with concentrated sulfuric acid, conversion of nitrogen to ammonium sulfate, and subsequent measurement of the ammonia produced. The protein content is then determined using the total nitrogen content.

### 2.4 Texture properties

Texture traits of all the cheese samples were determined using a Texture Analyzer (XT2i, Stable Micro Systems, Ltd., Godalming, Surrey, UK) with a Warner–Bratzler shear device [50 Newton (N) load cell; 2 mm/s crosshead speed]. For each cheese, 1 cylinder-shaped core sample was taken (1 cm^2^ cross sectional area; 3 cm long). Texture data were reported as hardness (defined as the maximum shear force, expressed in N), adhesiveness (describes the work needed to overcome attractive force between food and other surfaces, expressed in N/s), resilience (which referred to the degree to which the cheese regains its original shape during the biting process, expressed in %), cohesiveness (the tendency of cheese to remain together, and resist breaking into several pieces, during compression), springiness (a measure of ability that the deformed cheese returned to the initial position after the removal of the force, expressed in %), gumminess (the energy required to disintegrate a semi-solid food to a state ready for swallowing, expressed in N), chewiness (the work needed to masticate a solid food to a state ready for swallowing, expressed in N/s), respectively.

### 2.5 Chemometric analyses

#### 2.5.1 Editing of the spectra, data fusion and Bayesian models

Before data fusion and spectra analysis, each instrument’s raw absorbance values of each wavelength of the spectra were centered and scaled to a null mean and a unit variance. Then, samples having a large spectral distance (i.e., Mahalanobis distance >3) were considered outliers and removed from the calibration dataset. After scaling, the NIR and Raman spectra were concatenated and stored as single matrix to be used for the spectra analysis. This is called low-level data fusion (18, 19). No other mathematical preprocessing was applied to the spectra. The matrix comprises m-rows (number of individual samples) and n columns (measurement variables from each source). In this study, fusion data comprised a total of 2,000 variables with the Raman and NIR instrument contributing for 1,600 and 400 wavelengths, respectively.

The calibration models built using the Bayesian approach (15) were developed by using the Bayesian Generalized Linear Regression [BGLR; (20)] package, available in R software (21). Each trait was regressed to a new pool of wavelengths using the following equation:

$$y=\mu +\overset{t}{\underset{j\;}{\sum }}x_{ij}\beta_{j}+e_{i}$$

Where μ is the overall mean, x_ij_ are the NIR, Raman or fused spectra’s x axis, i is the sample (from 1 to 54), t is the number of wavelengths of the studied spectra (400 for NIR and 1,600 for Raman), and j the wavelengths (950 to 1,650 nm for NIR, and from −50 to 4,000 rel cm^−1^ for Raman), β_j_ are the regression coefficients, and e_i_ is the residual assumed to be independently and identically distributed with a normal distribution with mean equal to 0 and variance equal to s^2^_e_. The Bayes B model implemented in the package we used incorporates prior information about the model parameters and updates this information based on the observed data to estimate the effects of wavelengths on the phenotypes (15).

#### 2.5.2 Editing of the spectra and PLSR models

PLSR models were performed with the software Unscrambler (Aspen Tech, MA, USA). For the classification of the seasoning and PDO type of each cheese sample, PLS-DA models have been performed by Solo, version 9.2.1 (Eigenvector Research, Inc., Wenatchee, WA, USA). The different parameters were obtained from the different reference methods and were used as reference values for the model.

For the PLSR and PLS-DA model construction, from the total 54 spectra, 36 were selected by the Kennard-Stone (21) algorithm and used as calibration samples and the following 18 were kept as validation set. By doing this, the final model will have the maximum spectral variability to reach the best prediction capability.

The spectral pretreatments used to construct the PLSR and PLS-DA models included standard normal variate (SNV) (22), used to correct baseline shifts and variations in intensity across spectra; Savitzky–Golay Derivatives with second-order polynomial fitting (1st D and 2nd D) (23), to reduce high-frequency noise in a signal due to its smoothing properties and to reduce low-frequency signal (e.g., due to offsets and slopes) using differentiation and the second-order derivatives to highlight spectral features; smoothing (moving average, MA) (24); linear baseline correction (BLC) (25) and orthogonal signal correction (OSC), used to remove unwanted variations in the X- data that are unrelated to interest response (Y) (26). The selected pretreatments allowed the reduction of multiplicative effects derived from the physical characteristics of the samples and allowed the enhancement of the differences between spectra that will allow the performance of the desired classifications and quantitative models. The MA and BLC were necessary only for Raman spectra, as MA was useful to reduce the noise, whereas BLC corrected the curvature caused by the fluorescence effect.

#### 2.5.3 Cross-validation models

##### 2.5.3.1 Bayesian approach

For the classification of the seasoning and PDO type of each cheese sample and the prediction of the chemical composition and texture traits, a random cross-validation was applied, in which 80% of the total records were randomly selected and used to build the equation (calibration set; CAL), and the remaining 20% of records were used to test the model (validation set; VAL). To account for sample variability, the procedure was repeated 10 times for the classification and 5 times for the quantification. The results were averaged over the replicates. The standard deviation (SD) across the replicates was also calculated. The coefficient of determination (R^2^_VAL_), the root mean squared error of validation (RMSE_VAL_) and relative error of prediction (RSEP%) were used to assess the models’ performances. As the Bayesian approach uses a linear regression model, it was necessary to establish a decision criterion for interpreting the predicted values. For the prediction of the PDO, the label were switched to numerical value (0 for GP and 1 for PR) and a critical threshold was set up at 0.5. All predicted value <0.5 are attributed to GP while values >0.5 are classified as PR. For the prediction of the seasoning time, the model used the numerical value in months and the predicted values were divided in 3 classes: “young” (<17 months), “mid” (17–28.5 months) and “old” (>28.5 months). The ranges were decided in order to distinguish the 3 seasoning times and allowing the comparison between PDO (as their seasoning time was not the same). The percentage of attribution of each class was also calculated to assess the model’s accuracy.

##### 2.5.3.2 PLSR and PLS-DA approaches, and data fusion

Previously to any calibration or classification model an exploratory analysis was done to find the combination of pretreatments that provided better discrimination between PDO and ripening times classes.

For each instrument, prior to data fusion and spectra analysis, the raw absorbance values of each wavelength of the spectra were centered and scaled to a null mean and a unit variance. Then, samples having a large spectral distance (i.e., Mahalanobis distance >3) were considered outliers and removed from the calibration dataset. After scaling, the NIR and Raman spectra were concatenated and stored as a single spectra matrix that could be used for the spectra analysis. The matrix comprises m-rows (number of individual samples) and n columns (measurement variables from each source). Fusion data comprised a total of 2,000 variables, with the Raman and NIR instruments contributing for 1,600 and 400 wavelengths, respectively. Calibration models were constructed using the PLSR algorithm and internally validated by cross-validation (the leave-one-out method for PLSR and venetian blinds for PLS-DA). The coefficient of determination (R^2^_VAL_), the root mean squared error of validation (RMSE_VAL_) and relative error of prediction (RSEP%) were used to assess the models’ performances.

$$\mathrm{RSEP}\;\left(\%\right)=\sqrt{\frac{\sum_{i=1}^{n}{\left(y_{i}-{\hat{y}}_{i}\right)}^{2}}{\sum_{i=1}^{n}{\left(y_{i}\right)}^{2}}}\times 100$$

$$\mathrm{RMSEVAL}=\sqrt{\frac{\sum_{i=1}^{n}{\left(y_{i}-{\hat{y}}_{i}\right)}^{2}}{n}}$$

Where y_i_ is the reference value for validation set sample i, ŷ_i_ is the predicted value for validation set sample i, and n is the number of samples in the validation set.

The optimum number of latent variables was determined from a plot of the explained variance against the number of factors. Then, the initial model was refined by selecting those factors resulting in the lowest relative standard error for prediction (RSEP%), bias, and SD for the validation set.

For the classification, the percentage of attribution of each class was also calculated to assess the model’s accuracy.

Then, a permutation tests were conducted on the Bayesian and PLS models to assess their predictive performance and rule out potential overfitting to the spectral data’s inherent structure. The tests involved randomly shuffling the class labels (PDO and age) 500 times and refitting the models to each shuffled dataset. From this test we constructed a null distribution representing the range of outcomes expected under the assumption of no true relationships between the spectral data and the class labels. A key indicator of a model’s genuine predictive ability is its performance relative to this null distribution. If only a small percentage of the permuted datasets yields results comparable to or better than those of the original model, it suggests that the original model’s performance is unlikely to be attributable to chance associations or overfitting. Instead, it provides evidence that the model has successfully captured meaningful patterns in the spectral data that are genuinely predictive of the class labels (27). Since only 2% of the permutations yielded results comparable to or better than those of the original Bayesian models for all spectral matrices, and none of the permutations achieved results similar to the developed PLS model (data not shown), the results will not be further discussed, as the model actually predicted the class label (PDO or age) from the spectral data, without overfitting to a casual structure of the data.

## 3 Results and discussion

### 3.1 Prediction of seasoning and PDO type: Bayesian vs. PLS-DA across different type of spectra

Table 1 reports the descriptive statistics for composition traits and texture properties of the two types of PDO cheeses: PR and GP PDO cheeses. As regards to the chemical composition, the PR had a slightly higher moisture content (27.5%) compared to GP (26.7%), with similar CV (9 and 8%, respectively, for PR and GP). Moreover, PR contained more fat (33.7%) and less protein (34.3%) contents, compared to GP (30.1 and 32.4%, respectively, for fat and protein). Regarding texture properties, the hardness of both cheeses was similar, with PR having a mean hardness of 18.38 N and GP of 18.33 N. The CV values were relatively high, indicating some variability within each cheese type (15 and 19%, respectively) probably due to the wide range of seasoning of the analyzed samples. Adhesiveness refers to the ability of a cheese sample to stick or adhere to surfaces. Parmigiano Reggiano showed higher adhesiveness (−0.76 N/s) compared to GP (−0.47 N/s). Both had negative values, indicating that the cheese sample exhibited a low tendency to stick or adhere to surfaces. This can be a desirable characteristic for some types of cheese, like the hard ones. The CV was high (30 and 66%, respectively), which could be due to the different ripening times within each PDO cheese. Both cheeses exhibited similar resilience values (9.30 and 9.20%, respectively, for PR and GP) with higher CV for GP (9 and 14%, respectively). Cohesiveness quantifies the degree to which a cheese sample resists falling apart or fragmenting when it is bitten, chewed, or compressed. It provides insights into the cheese’s ability to maintain its integrity and internal structure during consumption, and from this point of view the two PDO had similar values (0.27 and 0.26, respectively, for PR and GP). As regards to springiness, this was higher in GP (54.8%) compared to PR (47.3%), with also higher CV (27 and 20%, respectively). Both cheeses had similar gumminess values (4.98 N and 4.87 N, respectively, for PR and GP), with higher CV for GP (23 and 28%, respectively). Grana Padano was also slightly chewier (2.82 N/s) than PR (2.45 N/s), with also higher CV (51 and 42%, respectively).

**Table 1:** Descriptive statistics (Mean ± SD, and CV) of composition and texture properties of Parmigiano Reggiano and Grana Padano cheese samples.

	PDO cheese			
	Parmigiano reggiano		Grana padano	
*Chemical composition, %*	Mean	CV	Mean	CV
Moisture	27.5(±2.6)	9	26.7(±2.1)	8
Fat	33.7(±1.3)	4	30.1(±2.9)	10
Protein	32.4(±3.0)	9	34.3(±2.0)	6
*Texture properties*				
Hardness, N	18.38(±2.8)	15	18.33(±3.4)	19
Adhesiveness, N/s	−0.76(±0.2)	30	−0.47(±0.0.3)	66
Resilience, %	9.30(±0.8)	9	9.20(±1.3)	14
Cohesiveness	0.27(±0.03)	11	0.26(±0.1)	19
Springiness, %	47.3(±9.4)	20	54.8(±14.6)	27
Gumminess, N	4.98(±1.2)	23	4.87(±1.4)	28
Chewiness, N/s	2.45(±1.0)	42	2.82(±1.4)	51

### 3.2 Prediction of seasoning and PDO type: Bayesian vs. PLS-DA across different type of spectra

Tables 2, 3 show the numbers of correctly and wrongly identified samples for the seasoning and PDO type by using the Bayes B model (Table 2) and the PLS-DA model (Table 3) with NIR, Raman and fused (NIR + Raman) spectra, respectively. Regarding the seasoning and PDO identification (Table 2), the NIR technique performed relatively well with accuracy ranging from 75% (Young) to 90% (Mid) and with a correct % of identification of 69% for GP and 59% for PR. The Raman performed poorly on the seasoning time, with a correct identification of 12 and 17%, respectively, for young and old ripening times, but achieved better results for mid seasoning (83%). The exact reasons for the observed performances can be multifaceted. However, some potential factors contributing to the poor results for young and old ripening times, when using a Bayesian approach, could include the dataset size, as in a small dataset, the observed information may not be comprehensive enough to fully capture the complexity of the relationship between Raman spectra and the ripening times of cheese. For the PDO identification, Raman achieved 83% accuracy for GP and 78% for PR. Regarding the results from the data fusion, this is the first work the authors are aware of, presenting results about data fusion on the prediction of PDO type and seasoning time. The spectra fusion provided consistent results with accuracies from 64 to 77% for the seasoning time, and high accuracy for both GP and PR (89%). As regards to PLS-DA used for the seasoning identification (Table 3), NIR achieved relatively high accuracy, ranging from 66 to 88%. For the PDO identification, NIR achieved perfect accuracy (100%) for GP and high accuracy (88%) for PR. Raman exhibited moderate accuracy, ranging from 50 to 75% for the seasoning time, but it achieved 100% accuracy for both PDO cheeses. The fusion of the spectra provided good accuracy, from 66 to 83% for the identification of the seasoning time, and was excellent for GP (100%) and good (77%) for PR. Comparing the two chemometric approaches, the Bayes B Model achieved very good accuracy for some categories within seasoning and PDO type but overall, the PLS-DA models consistently outperformed the Bayes B in terms of accuracy across all the instruments (NIR, Raman, and fused spectra). In particular, PDO identification using the PLS-DA models showed very high accuracy, with GP being correctly identified at 100% in all three type of spectra. As reported by Silva et al. (28), the chemometric methods most commonly used for the differentiation between cheese samples by origin, were mainly linear discriminant analysis, principal component analysis and the PLS-DA methods. For example, Karoui et al. (29) reached a 91% classification rate to determine the geographic origin of Gruyère PDO and L’Etivaz PDO Swiss cheeses. Yet again, in the study by Ottavian et al. (30), the use of PLS-DA demonstrated its value in differentiating NIR spectra in relation to the production period of Asiago d’Allevo cheeses, yielding 100% correct classification. A result that is comparable to that attained with the direct assessment of chemical properties. On the contrary, the Bayesian methods have not yet been applied to the infrared spectra of dairy products for the authentication of cheeses or discrimination purposes. However, Li Vigni et al. (31) used a class-modeling applied to Raman spectra of Parmigiano Reggiano PDO cheese samples to discriminate it from other cheese types. Their preliminary results were promising, showing sensitivity and specificity of 100% for the test set. In the case of data fusion, comparing the results from the integration of the spectra from NIR and Raman and the individual techniques, it comes that, when using the Bayesian model, the fusion of the data increased the performances in the prediction of the early seasoning (<12 months) and the identification of the PDO type. For the mid and old seasoning, the data fusion did not improve (mid seasoning, 77% vs. 90 and 83%, respectively, for fused vs. NIR and Raman) or improved only partially (old seasoning, 17% for Raman) the correct % of identification (Table 2). When using PLS-DA, the data fusion had the best prediction accuracy for the mid and old seasoning, improved the prediction over Raman for the early seasoning, did not change for the GP, and reduced the correct % of identification for PR (Table 3). It is interesting to note that in some cases, the fusion was not helpful to the improvement of the prediction accuracy, and this could be due to the fact that NIR and Raman may capture similar information about the sample that can be redundant, which in turn does not provide additional information (32). Other studies applied NIR and Raman spectra fusion for food authentication in other matrices: Márquez et al. (33) tested two data fusion strategies (mid and high level) combined with a multivariate classification approach for the identification of adulteration of hazelnut paste with almond. Their sensitivity and specificity values were between 96–100% and 88–100% for the mid- and high-level data fusion strategies, respectively. Wang et al. (34) developed chemometric models using Vis–NIR and Raman spectral data fusion for the assessment of commercial infant formula storage temperature and time, with a RMSE_VAL_ of 0.7 for the mid-level data fusion to predict storage time at both 20° and 37°C. Again, Bragolusi et al. (19) in their preliminary study on the differentiation of Italian and Greek extra virgin olive oil, they merged NIR and Raman spectra by low and mid-level fusion and submitted to PLS-DA analysis. In cross-validation the accuracy was 94% for the low-level and 97% for the mid-level fusion.

**Table 2:** Identification of seasoning and PDO using the Bayes B Model with NIR, Raman, and Fused Spectra.

	Correctly identified	Wrongly identified	% of identification
NIR			
*Seasoning^1^*			
Young	26	8	75
Mid	27	3	90
Old	27	9	77
*PDO^2^*			
GP	37	17	69
PR	27	19	59
Raman			
*Seasoning*			
Young	4	30	12
Mid	25	5	83
Old	6	30	17
*PDO*			
GP	45	9	83
PR	36	10	78
Fused			
*Seasoning*			
Young	26	8	77
Mid	23	7	77
Old	23	13	64
*PDO*			
GP	48	6	89
PR	41	5	89

^1^Seasoning: young = 12 months; mid = 24 months; old = 36 months; ^2^ PDO: GP = Grana Padano; PR = Parmigiano Reggiano.

**Table 3:** Identification of seasoning and PDO using the PLS-DA Models with NIR, Raman, and Fused Spectra.

	Correctly identified	Wrongly identified	% of identification
NIR			
*Seasoning^1^*			
Young	7	1	88
Mid	4	2	66
Old	4	1	80
*PDO^2^*			
GP	10	0	100
PR	8	1	88
Raman			
*Seasoning*			
Young	3	2	60
Mid	6	2	75
Old	3	3	50
*PDO*			
GP	11	0	100
PR	0	8	100
Fused			
*Seasoning*			
Young	2	1	66
Mid	7	2	77
Old	5	1	83
*PDO*			
GP	10	0	100
PR	7	2	77

^1^Seasoning: young = 12 months; mid = 24 months; old = 36 months; ^2^ PDO: GP = Grana Padano; PR = Parmigiano Reggiano.

### 3.3 Prediction of chemical composition and texture properties: Bayesian vs. PLSR for NIR

Tables 4, 5 report the prediction statistics of the composition and texture properties of cheese deriving from the Bayesian and PLSR procedures using NIR spectra from the cheese samples. As regards the Bayesian approach (Table 4), the R^2^_CAL_ values for fat, protein, and moisture ranged from 0.11 (fat) to 0.50 (moisture), with quite high SD (from ±0.05 to ±0.13), suggesting substantial variability in the model’s performance. Indeed, the errors of calibration were also high (from 1.57 to 2.91% for RMSE_CAL_ and from 5.8 to 9.2% for RSEC%). Obviously, the results in the validation set were not accurate enough to be used at the dairy industry level, with R^2^_VAL_ < 0.50 within composition traits and high RSEP% (from 8 to 9%). Results from the texture properties were even worse, especially if we consider that the R^2^_CAL_ values were lower than the R^2^_VAL_ values. This fact could be due to the randomness in the data split (35) and/or the small sample size and/or to overfitting due to the complexity of the model (36). It is worth mentioning that the effectiveness of a chosen approach (Bayesian of PLSR in this study) depends on the specific characteristics of the data and the nature of the trait to be predicted. In some cases, Bayesian modeling with informative priors can provide valuable insights even with small datasets, as well as PLSR may be a more practical and less computationally demanding option. Indeed, results from the PLSR procedure (Table 5) generally showed better results than the Bayesian approach. Overall, the R^2^_CAL_ ranged from 0.37 (chewiness) to 0.89 (protein) with lower RSEC% (from 2.4 to 39%) compared to the Bayesian approach (RSEC% from 5.8 to 43%; Table 4). The R^2^_VAL_ values were good for moisture (0.69) and fat (0.63), with generally higher values compared to the Bayesian model, although for some traits, the R^2^_VAL_ was higher than the R^2^_CAL_ (adhesiveness). Comparing the two chemometric approaches, the Bayes B model using NIR spectra appeared to have some limitations in predicting both the composition and texture properties of cheese. The model’s performance varied across different traits, but with generally low R^2^ values and relatively high errors. This suggests that the model may require further refinement or the inclusion of additional features to improve its predictive accuracy for these cheese properties. In contrast, the PLSR procedure using NIR spectra appeared to be more effective in predicting both the composition and texture traits of cheese. However, these results may, in part, be attributed to the inherent constraints associated with the small sample size, underscoring the importance of considering the potential impact of limited data in interpreting the predictive capabilities of the model. There is an extensive amount of literature on the application of PLSR and NIR spectroscopy to the prediction of cheese chemical composition. In diverse kinds of cheeses and curds, all predictions of fat and the majority of predictions of protein and moisture/dry matter can be deemed excellent [*R*^2^ > 0.90; (37–39)]. NIR spectroscopy is, therefore highly suitable for predicting main chemical components like fat, protein, and moisture, and it has also been proved to be effective for monitoring the compliance of nutritional labels with EU tolerance limits of a wide varety of food products (40). However, it is not stable for predicting chemicals with small amounts in cheese products [e.g., minerals, fatty acids; (41)] since the amount of chemicals can alter the accuracy of prediction. NIR has a great degree of spectrum stability, which has made it quite popular at the industry level (42). However, literature related to NIR spectroscopy for cheese quality evaluation has become less in recent years (43) because applications for evaluating other attributes are inadequate, and innovative chemometric methods are not extensively used in research.

**Table 4:** Prediction statistics (Mean ± SD of the 5 replicates) of composition and texture properties of cheese deriving from the Bayesian model Cross-Validation procedure using NIR spectra from paste cheese samples.

*NIR*	Composition				Texture					
	Fat	Protein	Moisture	Adhesiv.	Chewiness	Cohesiv.	Gumminess	Hardness	Resilience	Springiness
Prediction statistics^1^										
R^2^_CAL_	0.11 ± 0.05	0.39 ± 0.10	0.50 ± 0.13	0.22 ± 0.05	0.16 ± 0.09	0.01 ± 0.21	0.11 ± 0.05	0.08 ± 0.03	0.20 ± 0.16	0.26 ± 0.14
RMSE_CAL_	2.91 ± 0.33	2.18 ± 0.15	1.57 ± 0.24	0.28 ± 0.01	1.05 ± 0.19	0.03 ± 0.01	1.11 ± 0.12	2.58 ± 0.48	0.95 ± 0.15	10.56 ± 1.60
RSEC%	9.20 ± 2.0	6.60 ± 0.5	5.80 ± 0.9	43.4 ± 1.7	41.4 ± 7.3	12.3 ± 3.1	23.0 ± 2.6	14.3 ± 2.7	10.4 ± 1.7	21.0 ± 3.2
Slope	−1.00 ± 1.96	0.33 ± 0.52	0.57 ± 1.15	0.23 ± 2.19	0.01 ± 3.07	1.73 ± 3.80	−0.72 ± 2.47	−2.13 ± 2.92	−0.97 ± 1.74	0.93 ± 2.27
R^2^_VAL_	0.12 ± 0.15	0.25 ± 0.32	0.47 ± 0.31	0.24 ± 0.21	0.37 ± 0.23	0.59 ± 0.29	0.25 ± 0.33	0.29 ± 0.32	0.34 ± 0.21	0.54 ± 0.26
RMSE_VAL_	2.89 ± 1.31	2.58 ± 0.71	2.41 ± 0.82	0.18 ± 0.07	1.50 ± 0.59	0.06 ± 0.03	1.56 ± 0.48	3.61 ± 1.31	1.51 ± 0.49	13.52 ± 5.86
RSEP%	9.10 ± 4.1	7.80 ± 2.1	8.90 ± 3.0	28.9 ± 11.1	58.8 ± 23.3	20.6 ± 9.3	32.3 ± 9.9	20.0 ± 7.3	16.4 ± 5.3	26.9 ± 11.7
Bias	−0.68 ± 3.12	0.69 ± 2.63	0.64 ± 2.50	0.00 ± 0.20	0.55 ± 1.53	0.02 ± 0.06	0.70 ± 1.50	1.47 ± 3.60	0.34 ± 0.21	0.54 ± 0.26

1 R^2^_CAL_ and R^2^_VAL_, coefficient of determination for the calibration and the validation ± Standard Deviation; RMSE_CAL_ and RMSE_VAL_, Root Mean Square error for the calibration and validation ± Standard Deviation; RSEC% and RSEP%, Relative error for the calibration and the validation ± Standard Deviation; Slope of the calibration equation ± Standard Deviation; Prediction bias, average difference between the predictions and the labels in dataset in absolute value ± Standard Deviation.

**Table 5:** Prediction statistics of composition and texture properties of cheese deriving from the PLS regression procedure using NIR spectra from paste cheese samples.

*NIR*	Composition			Texture						
	Fat	Protein	Moisture	Adhesiv.	Chewiness	Cohesiv.	Gumminess	Hardness	Resilience	Springiness
Prediction statistics^1^										
R^2^_CAL_	0.86	0.89	0.70	0.42	0.37	0.47	0.67	0.52	0.42	0.55
RMSE_CAL_	1.09	0.88	1.24	0.23	1.12	0.03	0.81	2.14	0.82	8.06
RSEC%	3.4	2.4	4.6	34.2	39.6	11.4	22.7	12.0	9.0	16.0
Slope	0.86	0.89	0.70	0.42	0.37	0.47	0.67	0.52	0.42	0.55
R^2^_VAL_	0.63	0.33	0.69	0.44	0.17	0.29	0.14	0.20	0.18	0.49
RMSE_VAL_	1.68	2.12	1.37	0.23	0.76	0.03	0.94	2.79	0.89	10.26
RSEP%	5.4	6.7	4.99	37.5	27.6	11.5	18.8	14.00	9.4	17.9
Bias	0.03	−0.08	0.28	−6.55	−17.25	0.01	340.34	−844.69	−0.09	−0.08

1 R^2^_CAL_ and R^2^_VAL_, coefficient of determination for the calibration and the validation; RMSE_CAL_ and RMSE_VAL_, Root Mean Square error for the calibration and validation; RSEC% and RSEP%, Relative error for the calibration and the validation; Slope of the calibration equation; Prediction bias, average difference between the predictions and the labels in dataset in absolute value.

### 3.4 Prediction of chemical composition and texture properties: Bayesian vs. PLSR for Raman

Tables 6, 7 report the prediction statistics of composition and texture properties of cheese deriving from the Bayesian and PLSR procedures, respectively, using Raman spectra from the cheese samples. As regards the Bayesian approach (Table 6), the R^2^_CAL_ values for composition traits ranged from 0.28 (protein) to 0.72 (fat) and were higher compared to the R^2^_CAL_ values obtained with the same procedure using NIR spectra (Table 4). The errors of calibration were still high (from 1.58 to 2.58% for RMSE_CAL_ and from 5.8 to 7.7% for RSEC%), but in the case of fat, the error was-3% lower than RSEC% of fat predicted with the same procedure applied to the NIR spectra. The results in the validation set were good, with R^2^_VAL_ ranging from 0.46 (moisture) to 0.74 (fat) within composition traits, although RSEP% were still high (from 5 to 9%). Fat percentage was the trait predicted at best with the highest R^2^_VAL_ (0.72) and the lowest RSEP% (5.3%). Indeed, it is acknowledged that fat molecules, which are primarily composed of carbon-hydrogen (C-H) bonds and carbon–carbon (C-C) bonds, exhibit characteristic vibrational modes that are readily detected by Raman spectroscopy. The vibrational frequencies of these bonds are unique to fats, providing a distinct Raman signature (44, 45). On the contrary, fitting statistics for the texture properties were poor, with R^2^_CAL_ values ranging from 0.19 to 0.43 and high calibration errors. Consequently, results in validation were not satisfactory, with R^2^_VAL_ values from 0.08 to 0.45. As aforementioned, this could be due to the randomness in the data split, the small sample size, and/or to overfitting due to the complexity of the model (35, 36). Moreover, compared to the same approach applied to the NIR spectra (Table 4), texture properties were worse predicted using the Raman spectra.

**Table 6:** Prediction statistics (Mean ± SD of the 5 replicates) of composition and texture properties of cheese deriving from the Cross-Validation procedure using Raman spectra from paste cheese samples.

*Raman*	Composition			Texture						
	Fat	Protein	Moisture	Adhesiv.	Chewiness	Cohesiv.	Gumminess	Hardness	Resilience	Springiness
Prediction statistics^1^										
R^2^_CAL_	0.72 ± 0.07	0.28 ± 0.06	0.64 ± 0.12	0.26 ± 0.08	0.38 ± 0.15	0.41 ± 0.10	0.29 ± 0.16	0.19 ± 0.07	0.41 ± 0.15	0.43 ± 0.07
RMSE_CAL_	1.89 ± 0.30	2.58 ± 0.10	1.58 ± 0.24	0.27 ± 0.02	1.02 ± 0.14	0.03 ± 0.01	1.08 ± 0.14	2.47 ± 0.40	0.97 ± 0.09	10.29 ± 0.68
RSEC%	5.9 ± 1.0	7.7 ± 0.3	5.8 ± 0.9	41.7 ± 3.0	40.3 ± 5.7	12.2 ± 2.0	22.4 ± 3.0	13.7 ± 2.2	10.5 ± 1.0	20.5 ± 1.4
Slope	0.12 ± 0.15	0.25 ± 0.32	0.47 ± 0.31	0.51 ± 1.59	5.89 ± 6.75	0.78 ± 3.54	−4.25 ± 3.47	−1.81 ± 2.05	−0.52 ± 7.65	2.58 ± 2.94
R^2^_VAL_	0.74 ± 0.17	0.50 ± 0.28	0.46 ± 0.31	0.18 ± 0.16	0.39 ± 0.35	0.16 ± 0.16	0.42 ± 0.37	0.08 ± 0.06	0.14 ± 0.12	0.45 ± 0.43
RMSE_VAL_	1.69 ± 1.11	2.49 ± 0.35	2.52 ± 0.81	0.22 ± 0.06	1.47 ± 0.47	0.05 ± 0.02	1.53 ± 0.50	3.61 ± 1.24	1.16 ± 0.22	12.27 ± 2.23
RSEP%	5.3 ± 3.5	7.5 ± 1.1	9.3 ± 3.0	35.1 ± 8.9	56.7 ± 18.5	19.8 ± 6.4	31.7 ± 10.3	20.0 ± 6.9	12.6 ± 2.4	24.4 ± 4.4
Bias	−0.97 ± 1.75	0.51 ± 2.53	0.58 ± 2.62	0.00 ± 0.27	0.53 ± 1.44	0.02 ± 0.05	0.61 ± 1.51	1.36 ± 3.62	0.09 ± 1.20	4.27 ± 11.97

1 R^2^_CAL_ and R^2^_VAL_, coefficient of determination for the calibration and the validation ± Standard Deviation; RMSE_CAL_ and RMSE_VAL_, Root Mean Square error for the calibration and validation ± Standard Deviation; RSEC% and RSEP%, Relative error for the calibration and the validation ± Standard Deviation; Slope of the calibration equation ± Standard Deviation; Prediction bias, average difference between the predictions and the labels in dataset in absolute value ± Standard Deviation.

**Table 7:** Prediction statistics of composition and texture properties of cheese deriving from the PLS regression procedure using Raman spectra from paste cheese samples.

*Raman*	Composition			Texture						
	Fat	Protein	Moisture	Adhesiv.	Chewiness	Cohesiv.	Gumminess	Hardness	Resilience	Springiness
Prediction statistics^1^										
R^2^_CAL_	0.86	0.59	0.93	0.64	0.99	0.82	0.88	0.96	0.96	0.88
RMSE_CAL_	1.15	1.82	0.62	0.19	0.14	0.02	0.43	0.54	0.19	4.30
RSEC%	1.1	4.8	2.3	30.3	4.8	6.2	8.5	2.9	2.1	8.0
Slope	0.86	0.59	0.93	0.64	0.99	0.82	0.88	0.96	0.96	0.88
R^2^_VAL_	0.52	0.48	0.48	0.25	0.59	0.63	0.25	0.51	0.49	0.63
RMSE_VAL_	1.75	1.89	1.50	0.25	0.83	0.03	1.08	2.61	0.84	7.92
RSEP%	5.4	6.2	5.6	33.6	29.6	9.8	21.6	14.2	9.1	15.7
Bias	0.28	−0.84	0.08	−0.40	57.79	0.01	117.33	83.68	0.04	−0.80

1 R^2^_CAL_ and R^2^_VAL_, coefficient of determination for the calibration and the validation; RMSE_CAL_ and RMSE_VAL_, Root Mean Square error for the calibration and validation; RSEC% and RSEP%, Relative error for the calibration and the validation; Slope of the calibration equation; Prediction bias, average difference between the predictions and the labels in dataset in absolute value.

Results from the PLSR procedure (Table 7) generally showed better results than the Bayesian approach. The R^2^_CAL_ ranged from 0.59 (protein) to 0.93 (moisture) with lower RSEC% (from 1.1 to 4.8%) compared to the Bayesian approach, as well as for texture properties, whose R^2^_CAL_ ranged from 0.64 to 0.99. Although the calibration statistics were satisfactory, the R^2^_VAL_ values were > 0.50, only for half of the traits examined. Compared to the fitting statistics using the same approach applied to the NIR spectra, the use of Raman spectra showed better results, both in terms of composition and texture properties and in general, also compared to the use of NIR and Raman spectra with the Bayesian models. Raman spectroscopy is less used than NIR spectroscopy, but it has gradually become a new analytical method for the structure identification of substances (46). In the dairy cheese industry field, Raman spectra were used to discriminate adulterated spreadable cheese with 100% accuracy and 95% confidence and to predict starch content with R^2^_VAL_ of 0.98 (12). Recently, Zhang et al. (47) combined Raman spectroscopy with a machine learning model for cheese product identification, with an average identification accuracy rate of 98%.

### 3.5 Prediction of chemical composition and texture properties: Bayesian vs. PLSR for fused spectra

There are no studies in the literature on integrating spectrum signals derived from various spectroscopic techniques or comparing different chemometric procedures at the dairy sector level. However, data fusion is a developing practice since collecting spectral data is becoming easier. The primary goal is to optimize the information gathered in order to take advantage of the synergies between the various types of information provided by diverse approaches (48). Data fusion can be done using low, mid, or high-level fusion techniques. Low-level data fusion involves the direct fusion (e.g., simple concatenation) of raw data from several sources. Tables 8, 9 report the prediction statistics of composition and texture properties of cheese deriving from the Bayesian and PLSR procedures, respectively, using the fused spectra (NIR + Raman) from the cheese samples. As also proved by the results above, different spectroscopic techniques can provide complementary information about a sample (e.g., chemical composition, structure). Therefore, integrating data sources from different spectroscopic techniques can provide a more comprehensive understanding of the cheese quality, and also increase the robustness of a prediction model (33, 49). As regards the Bayesian approach (Table 8), the R^2^_CAL_ values for composition traits ranged from 0.59 (protein) to 0.93 (moisture) and were much higher compared to the R^2^_CAL_ values obtained with the same procedure using NIR and Raman spectra (Tables 4, 6). The R^2^_VAL_ of composition traits improved with respect to the NIR prediction (0.41, 0.30, and 0.53 of fused vs. 0.12, 0.25, and 0.47 of NIR, respectively, for fat, protein, and moisture) and with respect to the moisture content predicted by using Raman spectra (R^2^_VAL_ = 0.46). Results in calibration for texture properties were also much more improved (R^2^_CAL_ ranging from 0.18 to 0.49) with also lower errors (RSEC% from 10 to 41%) compared to NIR spectra, whereas if compared to the Raman, the R^2^_CAL_ values were lower, with similar errors. In validation, some texture traits benefited from the fusion of the spectra from the two instruments (e.g., hardness and resilience), whereas others were better predicted with NIR (e.g., adhesiveness, cohesiveness, springiness) or Raman (e.g., chewiness, gumminess) only. So, the fusion of the two techniques (NIR and Raman) with the Bayesian model tended to have moderate calibration and prediction performance across most attributes, with some variation in the quality of results. This might be the result of the complementing information that NIR and Raman provide regarding the structure and composition of the cheese, as well as the possibility that the performance of the model is impacted by the variability in the cheese samples and the quality of the spectroscopic data. However, Raman spectra generally outperformed both fused and NIR spectra, with higher R^2^_CAL_ and R^2^_VAL_ values and lower RMSE_VAL_ and RSEP% for several attributes. The NIR spectra exhibited the lowest calibration and prediction performance compared to the other two types of spectra.

**Table 8:** Prediction statistics (Mean ± SD of the 5 replicates) of composition and texture properties of cheese deriving from the Cross-Validation procedure using fused NIR and Raman spectra from paste cheese samples.

*Fused*	Composition			Texture						
	Fat	Protein	Moisture	Adhesiv.	Chewiness	Cohesiv.	Gumminess	Hardness	Resilience	Springiness
Prediction statistics^1^										
R^2^_CAL_	0.77 ± 0.13	0.58 ± 0.09	0.75 ± 0.11	0.32 ± 0.06	0.32 ± 0.07	0.39 ± 0.13	0.20 ± 0.08	0.18 ± 0.06	0.41 ± 0.09	0.49 ± 0.09
RMSE_CAL_	1.79 ± 0.30	1.96 ± 0.21	1.16 ± 0.26	0.26 ± 0.01	1.00 ± 0.18	0.03 ± 0.01	1.08 ± 0.13	2.47 ± 0.44	0.93 ± 0.13	9.31 ± 1.41
RSEC%	5.90 ± 1.0	7.70 ± 0.3	5.80 ± 0.9	40.8 ± 1.5	39.4 ± 7.2	11.6 ± 3.1	22.4 ± 2.7	13.7 ± 2.4	10.2 ± 1.4	18.5 ± 2.8
Slope	1.09 ± 0.96	0.53 ± 0.33	0.74 ± 0.70	0.35 ± 0.89	−1.10 ± 3.41	−0.13 ± 2.86	−3.26 ± 2.56	−4.75 ± 7.47	−2.73 ± 3.35	0.61 ± 1.49
R^2^_VAL_	0.41 ± 0.25	0.30 ± 0.33	0.53 ± 0.41	0.19 ± 0.19	0.29 ± 0.34	0.34 ± 0.43	0.39 ± 0.29	0.49 ± 0.45	0.57 ± 0.32	0.37 ± 0.29
RMSE_VAL_	2.58 ± 1.03	2.37 ± 0.54	1.83 ± 0.96	0.21 ± 0.04	1.48 ± 0.57	0.05 ± 0.02	1.58 ± 0.49	3.71 ± 1.25	1.35 ± 0.32	12.65 ± 4.80
RSEP%	8.10 ± 3.2	7.10 ± 1.6	6.70 ± 3.5	32.6 ± 5.8	58.1 ± 22.2	19.9 ± 8.6	32.6 ± 10.2	20.6 ± 6.9	14.7 ± 3.4	25.2 ± 9.5
Bias	−1.24 ± 2.51	0.52 ± 2.42	0.87 ± 1.87	0.02 ± 0.21	0.60 ± 1.48	0.02 ± 0.06	0.68 ± 1.53	1.39 ± 3.71	0.16 ± 1.40	5.24 ± 12.61

1 R^2^_CAL_ and R^2^_VAL_, coefficient of determination for the calibration and the validation ± Standard Deviation; RMSE_CAL_ and RMSE_VAL_, Root Mean Square error for the calibration and validation ± Standard Deviation; RSEC% and RSEP%, Relative error for the calibration and the validation ± Standard Deviation; Slope of the calibration equation ± Standard Deviation; Prediction bias, average difference between the predictions and the labels in dataset in absolute value ± Standard Deviation.

**Table 9:** Prediction statistics of composition and texture properties of cheese deriving from the PLS procedure using Fused Spectra from paste cheese samples.

*Fused*	Composition			Texture						
	Fat	Protein	Moisture	Adhesiv.	Chewiness	Cohesiv.	Gumminess	Hardness	Resilience	Springiness
Prediction statistics^1^										
R^2^_CAL_	0.82	0.86	0.54	0.77	0.39	0.62	0.19	0.42	0.53	0.50
RMSE_CAL_	1.24	1.05	1.58	0.14	1.00	0.03	1.03	2.17	0.74	9.32
RSEC%	3.9	3.2	5.3	20.9	33.9	9.8	21.9	11.9	8.9	17.2
Slope	0.82	0.86	0.54	0.77	0.39	0.62	0.19	0.42	0.53	0.50
R^2^_VAL_	0.42	0.15	0.19	0.21	0.02	0.22	0.02	0.02	0.23	0.07
RMSE_VAL_	2.14	2.17	2.08	0.31	1.24	0.03	1.31	3.64	0.99	10.78
RSEP%	6.7	6.5	7.6	46.4	49.8	11.2	25.6	18.9	10.5	22.4
Bias	−0.09	0.35	−0.04	40.2	452.66	−0.01	−655.20	−594.50	−0.22	3.99

1 R^2^_CAL_ and R^2^_VAL_, coefficient of determination for the calibration and the validation; RMSE_CAL_ and RMSE_VAL_, Root Mean Square error for the calibration and validation; RSEC% and RSEP%, Relative error for the calibration and the validation; Slope of the calibration equation; Prediction bias, average difference between the predictions and the labels in dataset in absolute value.

Results from the PLSR procedure (Table 9) generally showed good results but worse results compared to the Bayesian approach using the fused spectra. The R^2^_CAL_ values for composition traits ranged from 0.54 (moisture) to 0.86 (protein), although the RSEC% were better (from 3 to 5%) compared to the Bayesian approach. In validation, the accuracy of the PLSR was still worse than the Bayesian (R^2^_VAL_ from 0.15 to 0.42). The model for texture properties was better in calibration (R^2^_CAL_ from 0.19 to 0.77) than the Bayesian but with scanty predictive accuracy (R^2^_VAL_ from 0.02 to 0.23). Comparing the three techniques using the PLSR method, the results from the Raman spectra generally exhibited higher R^2^_CAL_ and R^2^_VAL_ values compared to fused and NIR spectra, indicating better calibration and predictive ability for most attributes. Raman spectra also had lower RSEC% and RSEP% values, suggesting better precision and accuracy in calibration and prediction. The NIR spectra showed moderate performance, with R^2^_CAL_ and R^2^_VAL_ values falling between those of Raman and fused spectra. For some attributes (e.g., fat, protein, moisture), NIR spectra outperform fused spectra regarding R^2^ and R^2^_VAL_ values. Adhesiveness, chewiness, and cohesiveness had relatively low R^2^_CAL_ and R^2^_VAL_ values in all three type of data, suggesting that they might be challenging to predict using these spectroscopic/chemometric techniques. In the scientific literature, the highest number of publications incorporating data fusion techniques is alcoholic beverages (27%) [e.g., (18)] followed by fruit and vegetables (17%) [e.g., (50)] and oils (13%) [e.g., (19)]. Milk and dairy products cover the smallest percentage (only 5%) (49), therefore, it is difficult to compare our results with the current literature. However, it is important to mention that simply combining data from various instruments (in this case, NIR and Raman) does not automatically improve prediction accuracy. For example, data fusion may not be advantageous if the single instruments produce extremely comparable information or if one of them introduces excessive noise. It is critical to examine the uniqueness of the information provided by each instrument and carefully consider whether the benefits of fusion outweigh the obstacles and potential drawbacks, such as redundancy and noise amplification (51, 52). This consideration becomes particularly pertinent in the context of a small sample size, where the potential benefits of data fusion must be carefully interpreted.

So, the most effective method was the PLS-DA approach applied to NIR for classifying seasoning time, and to Raman spectra for classifying PDO type. The NIR, coupled with the PLS technique, was the best one for chemical traits. With the exception of cohesiveness (Raman) and resilience (NIR/Raman) with a RSEP% lower than 10%. On the other hand, no chemometric strategy nor spectroscopic technique for predicting texture achieved good prediction levels to be employed in the dairy industry.

## 4 Conclusion

This proof of principle study provides new insights into the application of chemometric approaches for predicting the characteristics of GP and PR PDO cheeses. Specifically, it focused on the use of NIR and Raman spectra and their integration to achieve these predictions, as well as the potential to distinguish between the two PDO and their various ripening stages. As regards the classification models, the PLS-DA achieved the greatest results, correctly identifying the PDO type at 100%. The findings were enhanced by the data fusion in 60% of the cases using the Bayesian approach and 40% using the PLS-DA approach. As regards the prediction of chemical composition and texture traits, the Bayesian technique using Raman spectra for fat provided the greatest performance in validation. It is important to highlight that the accuracy of predictions did not consistently improve with data fusion. This suggests that the effectiveness of data fusion may vary depending on the specific analysis and the methods employed. It may be necessary to carefully consider when and how to apply data fusion in such studies. Mathematical spectra treatment before and after fusion may enhance prediction accuracy, especially when dealing with inherently distinct techniques. Moreover, larger dataset of high quality data is needed to improve the statistical power of the analysis. This can also help to validate the models and reduce the risk of overfitting.

## Data availability statement

The original contributions presented in the study are included in the article/supplementary material, further inquiries can be directed to the corresponding author/s.

## Author contributions

GS: Data curation, Investigation, Supervision, Writing – original draft, Writing – review & editing. LG-M: Formal analysis, Investigation, Resources, Writing – original draft. GD: Formal analysis, Writing – original draft. JS: Formal analysis, Funding acquisition, Writing – original draft. AM: Formal analysis, Investigation, Writing – original draft. VP: Resources, Writing – original draft. PB: Resources, Writing – original draft. GG: Formal analysis, Writing – original draft. CC-G: Conceptualization, Project administration, Writing – original draft.
